# Rhorix: An interface between quantum chemical topology and the 3D graphics program blender

**DOI:** 10.1002/jcc.25054

**Published:** 2017-08-31

**Authors:** Matthew J. L. Mills, Kenneth L. Sale, Blake A. Simmons, Paul L. A. Popelier

**Affiliations:** ^1^ Deconstruction Division Joint BioEnergy Institute Emeryville California; ^2^ Biomass Science and Conversion Technology Department Sandia National Laboratories Livermore Califonia; ^3^ Biological Systems and Engineering Division, Lawrence Berkeley National Laboratory Berkeley Califonia; ^4^ Manchester Institute of Biotechnology (MIB), 131 Princess Street, Manchester and School of Chemistry, University of Manchester, Oxford Road Manchester Great Britain

**Keywords:** quantum chemical topology, quantum theory of atoms in molecules, blender, molecular graphics, visualization

## Abstract

Chemical research is assisted by the creation of visual representations that map concepts (such as atoms and bonds) to 3D objects. These concepts are rooted in chemical theory that predates routine solution of the Schrödinger equation for systems of interesting size. The method of Quantum Chemical Topology (QCT) provides an alternative, parameter‐free means to understand chemical phenomena directly from quantum mechanical principles. Representation of the topological elements of QCT has lagged behind the best tools available. Here, we describe a general abstraction (and corresponding file format) that permits the definition of mappings between topological objects and their 3D representations. Possible mappings are discussed and a canonical example is suggested, which has been implemented as a Python “Add‐On” named Rhorix for the state‐of‐the‐art 3D modeling program Blender. This allows chemists to use modern drawing tools and artists to access QCT data in a familiar context. A number of examples are discussed. © 2017 The Authors. Journal of Computational Chemistry Published by Wiley Periodicals, Inc.

## Introduction

**Figure 1:**
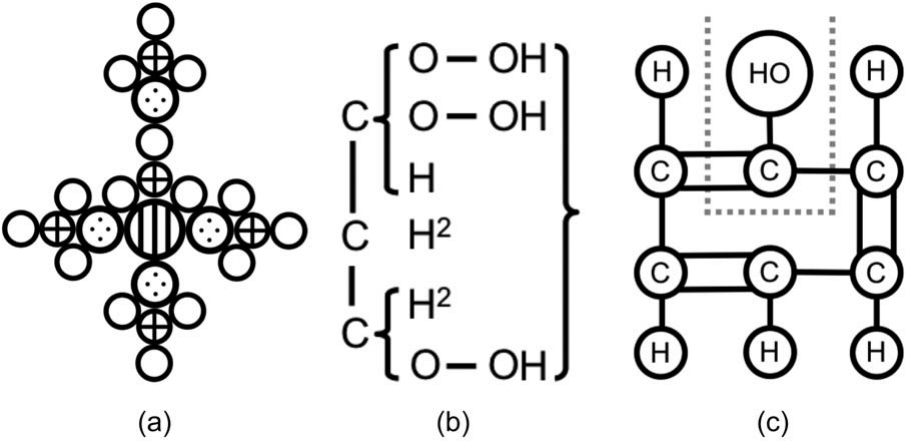
Recreations of early examples of molecular structure diagrams. a) Dalton's structure of alum (potassium aluminum sulfate). Empty circles represent oxygen atoms, crossed circles sulfur, dotted circles aluminum and the vertically lined, largest circle represents a potassium atom. b) Couper's 1858 structure of glycerine, the first to use solid lines to depict relationships between atoms. c) Crum Brown's 1867 structure of phenylic alcohol (phenol, based on Kekule's earlier formula).

Human understanding of chemistry has been advanced by visual and physical representations of chemical concepts since the emergence of the field from the alchemical tradition. In fact, it has been convincingly argued that chemists could not communicate or think about chemistry without the use of graphics,^1^ and modern chemistry relies on many different pictorial viewpoints.^2^ The most commonly cited first depictions of molecular structure based on a theory of chemistry are those of John Dalton, who, in “A New System of Chemical Philosophy” (1808), used circles inscribed with various shapes to represent different elements, and combined those circles to depict molecules of disparate complexity and their gases^3^ (see Fig. 1a).

**Figure 2:**
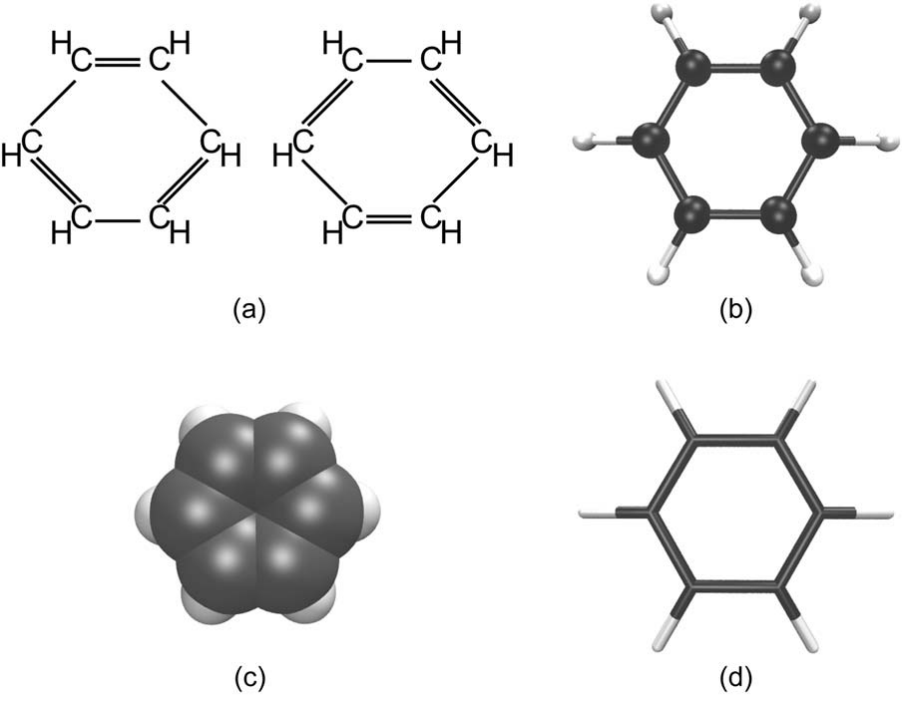
A series of representations of the structure of molecules in order of historical development. a) Recreation of Kekulé's original depiction of the cyclic structure of benzene, b) ball and stick (spheres at nuclear positions connected by cylinders) and c) Corey‐Pauling‐Koltun (overlapping spheres centered at nuclear positions) representations were invented as real‐world 3D models, while d) the line representation was initially necessitated by computer technology.

Despite the failings of its underlying theory, the spirit of this work was carried through in the writings of Berzelius, who replaced Dalton's cumbersome patterned circles with the now familiar (and then easily printed) alphabetical abbreviations.^4^, ^5^ Couper, Crum Brown, and Kekulé further advanced the notion of chemical structure via Frankland's concept of valence,^6^ Couper being the first to depict a bond between a pair of carbon atoms with a line^7^ (see Fig. 1b) and the latter famously deciphering the structure of the benzene molecule^8^ (Fig. 2a). Crum Brown developed a separate type of diagram with atomic symbols enclosed in circles^9^ (Fig. 1c). These foundational works defined the 2D representations of chemical systems still in common use today.

Prior to the advent of computers, 3D depiction of molecular structure for teaching and research was restricted to the use of real‐world objects. Before Fischer's 1891 work on isomerism in carbohydrates,^10^ 2D representations of 3D objects were uncommon. Physical models, however, can be found as far back as Hoffman's square‐planar ball‐and‐stick structure of methane in the 1860s.^11^ The ball‐and‐stick representation uses spheres for atoms, centered at nuclear positions, and uses straight connectors to depict bonds (see Fig. 2b). Subsequently, van't Hoff demonstrated the tetrahedral arrangement of bonds around carbon using cardboard models.^12^ Almost a century post‐Hoffman a complementary representation developed, which was based on the idea of the van der Waals radius,^13^ with atoms represented by large, space‐filling spheres without depiction of bonds. These models were developed by Corey and Pauling for the study of enzymes and other non‐catalytic proteins,^14^ and later improved by Koltun,^15^ resulting in the common name “CPK models” (Fig. 2c). Famous examples of structures discovered within this paradigm are the α‐helices^16^ and β‐sheets^17^ of proteins. Physical 3D models also played a central role in the discovery of the double‐helix structure of DNA.^18^, ^19^, ^20^

Prior to the rise of computational drawing of chemical species, a movement of artistic rendering flourished, focused on biochemistry. Pioneering crystallography work was accompanied by drawings, those of Geis appearing with the work of Kendrew^21^ and, most notably, those of Hayward included in the work of Pauling.^22^ These images made extensive use of both CPK and ball‐and‐stick models, both of which persist today, the former in its real world implementation as the building kits familiar to chemistry undergraduates, and both in their virtual implementation in computer graphics programs.

These representations have remained the standard in atomistic molecular representations as computer graphics have advanced. The representation of molecules as lines connecting bonded atoms is also very common (see Fig. 2d for a modern example), it being impossible to depict spheres with the first computers applicable to the problem, which relied on vector graphics.^23^, ^24^ Following early work, increasingly advanced software and hardware allowed more detailed and visually arresting computer rendering of chemical systems, especially as raster graphics permitted the CPK representation to be drawn on screens, and later enabled drawing of arbitrary molecular surfaces.^25^ As desktop computer systems became standard, user‐friendly software allowed any interested researcher to make images to assist in the completion and communication of their work. Pioneering software such as MolScript,^26^ Mage,^27^ and RasMol^28^ resulted in the ability to make computer graphics images of chemical systems, which so became part of the standard scientist's toolkit.

Despite the current proliferation of chemical drawing software, atomistic depictions of molecules and their assemblies still mostly rely on the representations shown in Figure 2. Of the common modern molecular graphics programs, VMD^29^ uses the line representation as its default, while PyMol,^30^ Jmol,^31^ Avogadro,^32^, ^33^ and GaussView^34^ use ball‐and‐stick as theirs. A casual survey of recent issues of chemistry‐focused journals reveals the near monopoly of both the ball‐and‐stick and line representations wherever atomistic, 3D images are used to communicate chemical results. By contrast, programs focused on macromolecules typically use surfaces or representations that show structure on a larger scale, such as the ribbon^35^, ^36^, ^37^ or cartoon depictions of the secondary structure of protein molecules. In such large systems, the greater detail of an atomistic representation can be lost in rendering, and important, coarser structure (e.g., the presence of helices and sheets in proteins) may not be apparent with a line representation. This communication focuses on those systems for which a detailed depiction of atoms, bonds, and interatomic interactions is illuminating. The development of other representations of large, biological systems is covered by Perkins.^38^, ^39^

### The physical basis of representations

Despite the wealth of examples littering the literature, 3D representations of chemical systems and related phenomena are often based on information that does not have a rigorous basis in theory, that is, properties that cannot be computed directly from first principles via an appropriately detailed quantum mechanical treatment. To give an example, the sticks of the ball‐and‐stick method depict bonds, but bonding still lacks a rigorous and unambiguous quantum mechanical definition, that is, there is no single agreed‐upon QM analysis that can determine whether a bond is present between two atoms. Indeed, the concept of chemical bond has been described as “too restrictive to account for the physics underlying the broad spectrum of interactions between atoms and molecules that determine the properties of matter.”^40^ In practice, empirical estimates of the “size” of atoms (another ill‐defined quantity in QM terms) are commonly used to determine whether the proximity of a pair of atoms is sufficiently small to warrant depiction of a bond between them. The CPK representation provides a second example, wherein each nucleus centers a sphere large enough to contain (practically) its entire associated atom. This representation neglects the fact that the spatial part of the wavefunction extends to infinity, and implies that atoms are overlapping spherical objects, which is not true in any quantitative theory of quantum chemistry.

Although representative of the problem at hand, the above two examples are by no means a complete list of popular representations and their connection to loosely‐defined empirical chemical concepts. Clearly, these representations have been of great utility in chemistry and have a strong historical basis, yet we are at a time where better methods can be used. To provide the *precise* understanding of chemistry necessary for exacting reasoning, visual representations must map to well‐defined, quantitative concepts.

### Quantum chemical topology

Quantum chemistry provides the source of well‐defined concepts that mirror the favored empirical concepts of chemists, for example bonding, isomerism, resonance, and so forth. However, the wavefunction‐centric view of chemistry is hampered by the massive dimensionality of these mathematical objects. A much more familiar concept is the scalar field defined in a 3‐dimensional real vector space, $R^{3}$, with which humans are certainly comfortable, knowingly or not. Thus, one possible redrawing of the problem is to attempt to define chemistry in terms of scalar fields computed from the wavefunction. This approach was first suggested by Schrödinger in the fourth of his series of six papers on Wave Mechanics^41^, ^42^ where the electron density was first defined; the fundamental role this quantity plays in molecular structure was noted soon after by London.^43^ This area of research has grown significantly, such that the umbrella term Quantum Chemical Topology (QCT) was coined^44^ in 2003, and several reviews have been published as (e.g., Popelier^45^, ^46^). QCT encompasses all approaches that use the mathematical language of dynamical systems (e.g., attractor, critical point (CP), gradient path, separatrix) to analyze scalar functions derived from the wavefunction. The canonical approach is the theory termed “Atoms in Molecules” (AIM) by its creator.^47^ This theory takes the topology of the electron density as its central focus, although all scalar fields have a topology and the abstractions described in the following apply to each. For example, other than the electron density,^48^ its Laplacian^49^, ^50^ and the electron localization function^51^ are commonly analyzed. A much more extensive list can be found in Popelier.^46^

**Figure 3:**
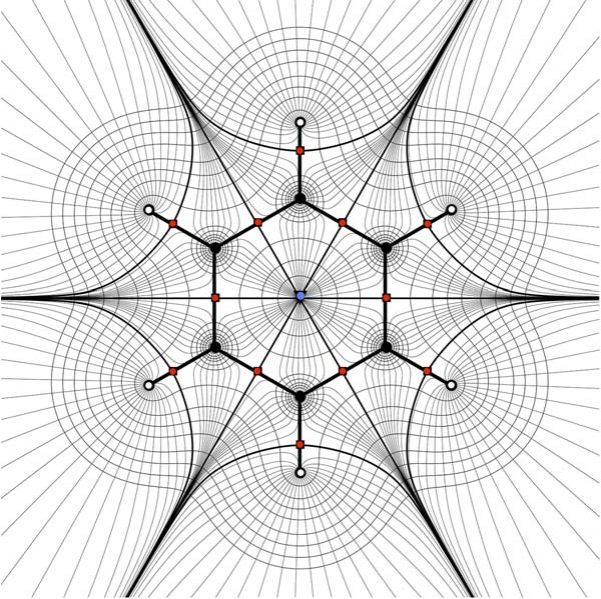
Rendering of the topology of benzene in its molecular plane. Red dots indicate the presence of bonds, and the blue dot indicates the presence of a bounding ring of bonds. Hydrogen nuclei are filled with white to distinguish them from carbon nuclei (black dots). Gradient paths (see Methods) are depicted with both heavy black lines (cf., Fig. 4a) and in light gray. Constant envelopes of electron density are also shown in light gray. [Color figure can be viewed at wileyonlinelibrary.com]

**Figure 4:**
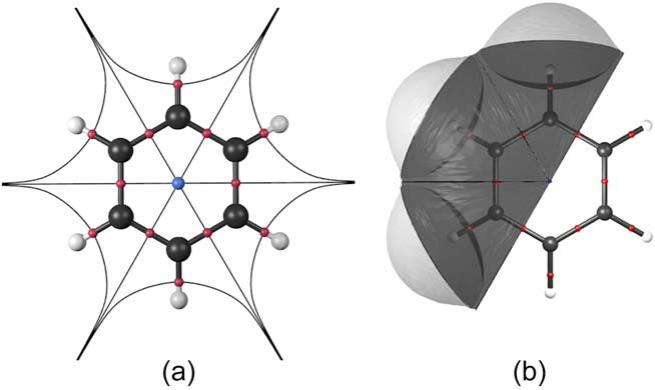
AIM‐determined analogues of a) ball and stick and b) CPK models (see Figs. 2b and 2c) drawn with Rhorix. The former depicts bonds with red spheres, and the ring with a blue sphere. Nuclei are represented with larger spheres, and paths connecting nuclei (here interpreted as chemical bonds) are colored according to the nuclear element. Other gradient paths in the molecular plane are depicted with thin lines and make clear the exhaustive partitioning of the system into 12 atomic basins. [Color figure can be viewed at wileyonlinelibrary.com]

A recent review of the state of QCT noted that the theory has “a visual appeal that has not been fully exploited.”^52^ When AIM was first described in the literature, the most common visualization found in publications was the 2D contour plot of the gradient vector field of a scalar function, as shown in Figure 3 for the molecular plane of the benzene molecule. Such images allow the location of regions of space associated with a given nucleus and of CPs, which reflect the structure of a molecule. Representation of trajectories traced out by the gradient gives a sense of the distribution of electron density within a system, while constant‐value envelopes communicate the rate of drop‐off of density away from nuclei. Despite their utility, only a 2D slice of the real 3D space can be depicted in a single such plot. Only planar systems submit to such an analysis; 3D systems require many such plots or the transition to 3D images. The latter has become the method of choice in current research. The same system is shown in Figure 4, where 3D information has been introduced to create representations similar to the ball and stick and CPK representations. Figure 4a shows the benzene molecule with nuclei now shown as spheres and gradient paths corresponding to bonds as cylinders. Figure 4b includes a representation of the atomic surface for 3 of the carbon atoms of the ring, mimicking the CPK representation. The generation of such figures is the focus of the remainder of this communication.

The original version of the authentic MORPHY program^53^ provided 3D visualization of topological elements by making use of the XMOL file format, which contains only the nuclei, specified by their element and Cartesian coordinates. Points in space associated with topological objects were represented as spheres by specifying them as tiny hydrogen nuclei. This approach was used to visualize the molecular graph^54^, ^55^ and interatomic surfaces,^56^, ^57^, ^58^, ^59^, ^60^, ^61^ (see Methods for definitions) and the same methodology has been used in visualization of the Laplacian of the charge density.^62^ Although pioneering at the time (and useful diagnostically) the resulting images left room for substantial improvement from an aesthetic point of view. A program allowing lines and surfaces to be drawn in an appropriate manner was clearly needed.

Improved visualization of atoms in 3D was made possible by the algorithm described in Rafat et al.^63^ This algorithm is able to generate smooth interatomic surfaces without gaps, and can deal with complex topological situations. This development occurred in tandem with completion of a GUI for MORPHY that utilized the Java3D application programming interface, now named IRIS. The MORPHY GUI has thrived, providing graphics for a long list of publications, including recent contributions.^45^, ^46^, ^64^, ^65^, ^66^ However, this GUI does not provide much flexibility for rendering because much of the functionality of Java3D is not exposed to the user. For example, the graphical abstract of Ref. [ 67] required addition of a representation of the β‐sphere (a sphere centered at a nucleus and entirely contained by its associated atomic basin), which ultimately had to be achieved via alteration of the source code. In addition, the quality of the rendering is limited by the reliance of Java 3D (or currently, Java FX 8) on OpenGL. An alternative to IRIS is AIMStudio, the visualization component of AIMAll,^68^ a fast and heavily‐featured program for AIM analysis grown, through modification, from the code of the Bader group. This software allows depiction of the complete range of topological objects (for example gradient paths connecting bond critical points [BCPs] and ring critical points [RCPs]), and is also based on OpenGL, as is the recently released Topology Toolkit.^69^ VMD is able to read certain topological data files and is interfaced to rendering programs with more advanced capabilities than OpenGL. However, its approach to controlling the appearance of the rendered scene is not straightforward. Therefore, there is scope for an improved or new tool that overcomes these issues by providing access to advanced drawing and rendering programs.

### Improved rendering of QCT data

The focus of this communication is on defining and implementing an interface from QCT programs to state‐of‐the‐art rendering programs. Initial investigation of programs appropriate for generating topological images suggested that Maya and Blender would be suitable. Maya is a commercial program, and as its cost is significant, the proof‐of‐concept implementation described herein is written for Blender, which is free and open source and has a thriving associated online community. Furthermore, the modeling and rendering tools provided by Blender are far more advanced than those offered by OpenGL, and it will improve further as an industry tool under constant development. The remainder of this communication describes the abstraction of the topology of a scalar function, the mapping of its members to 3D objects, and the rendering of those objects with Rhorix/Blender.

## Methods

### Topology of a scalar function and its object‐oriented representation

**Figure 5:**
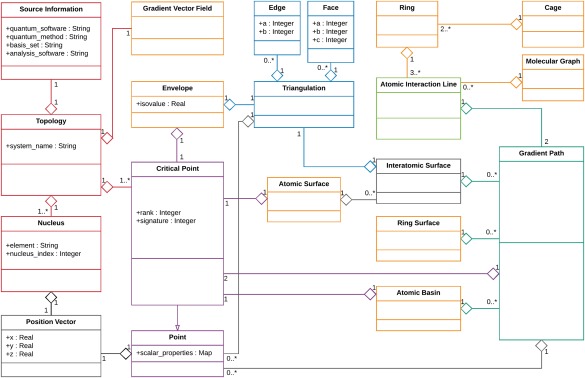
UML class diagram showing the static structure of the objects determined by a topological analysis of the electron density of a chemical system. Each rectangle represents a class (named in the top compartment). Middle and lower compartments list attributes and operations respectively. Connecting lines between classes define interclass relationships. Solid lines with a closed, unfilled arrowhead denote inheritance, for example, Critical Point is a subclass of the Point superclass. Aggregation (read as “has a”) is shown with a hollow diamond on the containing class and a line to the contained class. The number of objects of a given type is specified for each aggregation relationship. Note than connections from the Gradient Vector Field object are not depicted; children are shown in orange instead. [Color figure can be viewed at wileyonlinelibrary.com]

The scalar functions of QCT are expressed in terms of the wavefunction $\psi \left(r,R\right)$, usually computed under the Born‐Oppenheimer approximation (i.e., for a fixed nuclear configuration R) using the LCAO‐MO method with either Gaussian or plane‐wave basis sets. The nuclei (atomic numbers and positions) and number of electrons are input to a quantum chemistry method which determines the wavefunction. The scalar functions, however, are agnostic as to how the wavefunction was computed or is represented. Provided the combination of computational method and basis set corresponds to a sufficiently accurate model chemistry, the same topology of any given scalar function should be recovered in all cases. The central focus of AIM is the electron density, $\rho \left(r;R\right)$, a scalar field defined over the 3‐dimensional real space in which the chemical system of M electrons and N nuclei resides. Given the parametric dependence on R, it is usually omitted from the notation, a convention adopted in the foregoing. The electron density is a function that maps vectors $r\in R^{3}$ to scalars ρ∈R. The remainder of this communication focuses on this particular function, as it provides us with a computable concept of an atom in a molecule, as well as a quantitative concept of bonding and of structure. Representations of the topology of the electron density reveal information about both of these concepts. In this section, an abstraction of the topology of a scalar function (focused on the electron density) is described; the abstraction is depicted via a uniform modeling language (UML) class diagram (Fig. 5, see caption for key). Despite the focus on the electron density, the discussion applies in general to scalar functions and it is important to note that Rhorix makes no distinction as to the nature of the scalar field that produced a given topology. Thus, the software may be applied equally to any scalar field of interest provided its topology is stored in the appropriate format.

The basic object of interest is a Topology (red, upper left), unique for a given nuclear configuration and quantum chemical level of theory. Although not necessary for depiction of the topology of a given scalar function, the method, basis set, and program used to compute the wavefunction are associated with the Topology as a Source Information object. This enhances the reproducibility of the data used to draw an image. The name of the topological analysis software is also stored. The nuclei are of interest in drawing, particularly their Cartesian coordinates (abstracted herein as a Position Vector object, containing an array of real numbers of size 3) and elements, and are stored as a set of Nucleus objects (left, red), each of which has a single Position Vector, and a String giving the element (although an integer atomic number would be equally useful).

Points in the $R^{3}$ space can be represented with a class, Point (bottom class of second column in Fig. 5), whose first member is a single Position Vector. The electron density is a scalar defined at each point in space, and the Point class should reflect this. As noted previously, other scalar fields defined for $R^{3}$ may be of interest in depicting a chemical system. The other member of the Point class is, therefore, a map from strings (names of scalar functions) to real numbers (scalar values at this point) implemented with a hash table.

The topological properties of a scalar field can be completely specified by giving the number and type of CPs, points with position vector denoted $r_{cp}$, where $\nabla \rho \left(r_{cp}\right)=0$, that is, the gradient vanishes. Note that at an infinite distance from a nucleus this property is satisfied, thus the infinite vector is formally a CP. The type of a CP can be defined by two values, its rank and signature, which depend on the three eigenvalues of the Laplacian evaluated at the CP, $\nabla^{2}\rho \left(r_{cp}\right)$. The rank, $\omega \in \left\{0,1,2,3\right\}$, is the number of non‐zero eigenvalues, while the signature, σ, is the sum of their algebraic signs (i.e., working with −1 for the minus sign and +1 for the positive sign) whose allowed values depend on ω. These values are often written as the ordered pair $\left(\omega ,\sigma \right)$. The topology of chemical systems is dominated by CPs with ω=3, which appear in various chemical contexts, and have $\sigma \in \left\{-3,-1,+1,+3\right\}$. Each of these types is also given a name due to their correspondence to physical phenomena (examples can be found in the final section). Nuclear attractor critical points (NACPs) are maxima found nearly coincident to every nucleus of the system and have rank and signature $\left(3,-3\right)$. BCPs are saddle points appearing between pairs of atoms normally considered to be bonded through chemical intuition, and have rank and signature $\left(3,-1\right)$. RCPs are found when a set of atoms forms a ring and are denoted $\left(3,+1\right)$. Finally cage critical points (CCPs) are located when a set of connected atoms enclose a 3D area, and are denoted $\left(3,+3\right)$. CPs with ω≠3 are referred to as “degenerate” in general. CPs are inherently point objects, thus they form a subclass of the Point class (shown with a purple, open arrow in Fig. 5) with two additional members, integers storing the rank and signature.

The remaining objects of interest are features of the gradient vector field of the charge density, $\nabla \rho \left(r\right)$. The gradient vector field is the complete set of gradient paths of the system, where gradient paths are trajectories that connect pairs of CPs (of which one may be at infinity). These objects (and sets thereof) make several chemical concepts concrete. A Gradient Path object contains two CP objects, and a list of Point objects comprising a subset of the complete set of points on the trajectory chosen to allow a sufficiently smooth curve to be generated for rendering (see the aqua box on the bottom right of Fig. 5). In practice, it is unusual to be concerned with gradient paths as individual objects, and as such they are used to build more complex objects in the model. This is emphasized by the large number of relationships depicted with plain ends terminating at this class in Figure 5. Some objects (introduced below) consist of an infinite number of gradient paths^70^ and must, therefore, be represented by a chosen subset thereof. Others, which are described first, involve a finite set of gradient paths. The Topology has a single Gradient Vector Field associated with it. The latter object is composed of objects yet to be described, namely a molecular graph and sets of rings and cages, a set of atomic surfaces, ring surfaces, atomic basins and envelopes.

The combination of CPs and gradient paths allow the definition of a first‐principles concept of molecular structure. The basic object is the Atomic Interaction Line; a pair of gradient paths sharing a BCP and with differing NACPs. The object definition is shown in light green in the upper right of Figure 5. For an equilibrium geometry, this same object is termed a bond path. The set of atomic interaction lines of a system constitutes its molecular graph. The corresponding class, Molecular Graph, has as its only member a list of Atomic Interaction Line objects (which themselves contain all of the CPs connected by the molecular graph). The molecular graph contains the structure (connections between nuclei) of the chemical system.

There are two sub‐graphs of the molecular graph that are of interest, corresponding to rings and cages. A ring is present whenever a $\left(3,+1\right)$ CP is located in the topology. The RCP is connected directly to a set of BCPs via gradient paths termed “ring paths.” These BCPs are connected through atomic interaction lines to nuclei which bound the RCP in a ring. The Ring object is the set of (3 or more) Atomic Interaction Line objects and the RCP they enclose. Cages occur when a $\left(3,-3\right)$ CP is present in the topology. To exist, they require the presence of (2 or more) rings which fully enclose a region of $R^{3}$, and as such the Cage object is a set of Ring objects.

Besides molecular structure, analysis of the gradient vector field of the charge density also allows the definition of an atom within a molecule. An atom can be defined through the charge density in either of two ways, both of which may be of interest for depiction in various situations. Both rely on objects composed of an infinite set of gradient paths sharing a mutual start point, which can be represented pictorially using a subset thereof. The first, an atomic basin, consists of a set of gradient paths, which originate at an NACP and terminate at other CPs (most often infinity). An Atomic Basin object, therefore, has a list of gradient paths, all with a single NACP in common and terminating at a different CP (many such paths extend to infinity, and in practice must be truncated to lengths where the value of $\rho \left(r\right)$ exceeds some threshold).

The electron density is naturally partitioned into the aforementioned atomic basins by non‐trivial interatomic surfaces. An interatomic surface consists of a set of gradient paths that begin (when traced in the reverse direction) at a mutual BCP and end at infinity or at an RCP or CCP. The Interatomic Surface class is, therefore, a list of Gradient Path objects sharing a single BCP. Such surfaces form the boundaries between atoms, and points on their gradient paths belong to no single NACP. As BCPs are connected to a pair of nuclei, an IAS can be assigned to a pair of nuclei. The concept of an atomic surface provides the definition of the complete boundary of a given atom (with a single nuclear attractor). The union of all interatomic surfaces containing a BCP connected to the same NACP provides the surface of the atom containing that NACP. Thus, interatomic surfaces are not directly included in the Gradient Vector Field object and are instead included through Atomic Surface objects (see central box of Fig. 5).

A further class definition is necessitated by the fact that an atom that is not bound in all directions extends to infinity. In representing chemical systems, it is typical to depict a molecular outer boundary. To achieve an operational solution to this problem, isosurfaces are used. These are sets of points that share a common value of $\rho \left(r\right)$, and when gradient paths are traced in reverse from them (paths that are members of the atomic basin) they all connect to the same nucleus. The most typical value used to generate an isosurface is $\rho \left(r\right)=0.001$ au, widely quoted as the value that results in an atomic surface which encloses 98% of the system's electron density. The isosurface is abstracted as an Envelope, which has an associated isovalue. The points on the surface are included through a triangulation of the surface.

Depiction of surfaces as a set of gradient paths is not common in the literature. It is more usual to triangulate the set of points on the known paths to draw a single surface object. This requires the inclusion in the class diagram of a class named Triangulation (blue, upper center of Fig. 5). This class requires a set of points which are to be connected, and a representation of their connections. Blender requires either edges (a pair of points to be connected) or faces (a trio of points to be connected) and the Triangulation class, therefore, can contain any number of each.

### The XML‐based topology file

The UML class diagram of Figure 5 summarizes the general abstraction of the mathematical objects that may be depicted in a rendered image. The definition of a general file format with a document model reflecting Figure 5 is also warranted. The MORPHY GUI uses the .mif filetype, which is defined implicitly by its topological analysis and rendering programs. This fact has the negative result of making the filetype hard to parse, even with a copy of the relevant source code. A tagged plaintext file format is used by AIMAll, which does not allow for rigorous filetype definition. In contrast, the extensible markup language (XML) provides the means for definition of a document model (which defines which documents conform to the language) that can be shared between multiple programs without the need for complex parsers, creating a single encompassing standard. This defines the set of allowed elements, and a content model for each.

The description of the previous section provides the object hierarchy needed to define the filetype. The data model described by the UML diagram has been converted to an XML document type definition (Topology.dtd, provided with Rhorix), which allows automated checking of the conformity of files to the document model standard within Rhorix, and within a program written in any language that supports XML parsing. The Topology.dtd file is used in the program to validate input files before attempting to import them to Blender. Conversion of output from the disparate output formats of QCT codes into an XML format with a dictated form reduces the possibility of errors, both in the Python code and in any programs written to convert the output of scalar function analysis codes to rendering format. The topological model describes the topology of any scalar function, and Rhorix is, therefore, not restricted to depiction of the topology of the charge density.

### Mapping topology to 3D objects

The topological elements described above correspond to objects that can be mapped to chemical ideas, in particular atoms within molecules and molecular structure. These are to be represented in three dimensions to provide insight through visual depiction. To achieve this requires an explicit mapping from abstract mathematical objects (as elucidated in Fig. 5) to 3D shapes with various aesthetic properties. These aesthetics can be used to include more information in a rendered image, as well as to increase the visual appeal of rendered figures. While there is no single accepted mapping, a combination of historical standards, existing QCT practice and choices based on available data can be used to construct a possible canonical mapping, to which alterations can be made based on the particular results to be communicated. This mapping is implemented as a Blender Add‐On.

CPs, being single points in space, are typically represented as spheres in 3D. This choice allows CPs to be clearly located and provides two aesthetic variables for further discrimination between them: radius and material (which describes how objects interact with light, including their color). Those CPs corresponding to nuclei are sized relatively, according to van der Waals radius values of their elements. All remaining CPs are given smaller and equal radii, regardless of rank or signature. Due to the ubiquity of rank 3 CPs, each type is usually assigned a specific color for easy visual differentiation. Color is assigned to NACPs according to the element of the associated nucleus, and can be assigned to composite objects containing those CPs (see below). The remaining types of rank 3 CPs are colored by signature only. MORPHY uses purple, pink and red for BCP, RCP and CCPs respectively, while AimStudio uses green, red, and blue. Degenerate CPs are all given a single color different from the other CPs. Given their rarity (particularly in multiples) it will not be difficult for the user to set these colors appropriately.

All gradient paths can be represented as lines connecting the set of stored points on the trajectory. These may consist of a large number of points connected by straight lines, or alternatively can be smoothed by the use of Bezier curves, which is the approach used in Rhorix. Further information can be added to the visual representation by the appropriate choice of radius, material and design, which will be discussed for particular gradient paths forming different elements below.

The AILs comprising the molecular graph of a system are often divided into two types for mapping to 3D objects: those considered to connect bonded nuclei and those considered to correspond to a non‐bonded interaction (e.g., a hydrogen bond or H‐H interaction). Such a binary categorization of the AILs is not possible from first principles, and an empirical approach must be used. Those AILs corresponding intuitively to chemical bonds have typically been represented using a single radius for all, along with solid curves. The remainder, the so‐called “non‐bonded interactions” have either been represented using curves of different design (e.g., the dotted lines used in MORPHY) or by drawing curves with a smaller radius than used for bonds (as used in AimStudio). The color of the constituent gradient paths of an AIL is either uniform and dark (black or gray), or can be assigned based on the elemental identities of the NACP of each gradient path. The remaining individual gradient paths of interest are those that connect RCPs to BCPs (which can also be represented in AimStudio). The corresponding 3D curves can be given a unique color, or can be assigned the RCP color. In most images, they are not depicted. Most images of QCT calculation output only depict the molecular graph and atomic surfaces. However, the ability to depict rings and cages separately may assist in interpretation of the topology, hence their inclusion in the class diagram of Figure 5.

An atomic basin depicts the internal distribution of electron density within a single atom. This object contains only gradient paths originating at a single mutual NACP. Each individual gradient path can be represented as a curve, and each curve can be colored according to the NACP element. The radius of each curve is usually set constant. These depictions (for 3D systems) have been rare until recently.

Although the interatomic surface is composed of a set of gradient paths, as a surface it is more commonly depicted as a single object, which requires triangulation of a set of points on the surface. The same issue is faced when rendering constant value envelopes and a set of disconnected points is deemed inappropriate. An envelope can be directly connected with a single NACP. The atomic surface, as a union of interatomic surfaces is also associated with a unique NACP and can provide color, however if adjacent atoms (sharing a surface) are depicted at the same time, a single object will be depicted in two colors. An alternative is to color all interatomic surfaces identically. When solid atomic surface is drawn, they are often made transparent so that the molecular graph remains visible.

### Implementation of a Python add‐on for blender

A concrete implementation of the object model defined in the UML class diagram of Figure 5 is required for useful software. Python provides the necessary object‐oriented features for implementation of the Blender Add‐On. The class definition is a necessary element of Rhorix and follows Figure 5 exactly. It is not discussed here in detail but is provided in TopologyClasses.py within Rhorix.

**Figure 6:**
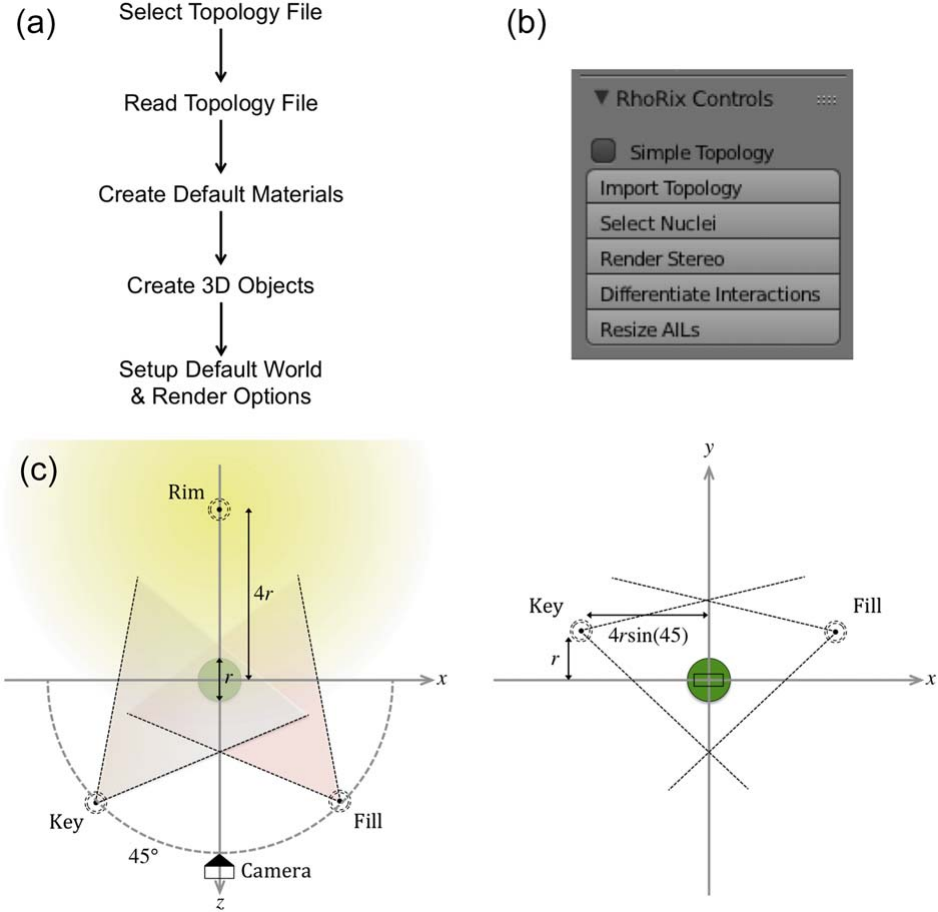
a) Rhorix Blender GUI control panel, b) Python add‐on execution flowchart and c) automatic 3‐point lighting setup diagram in the *xz*‐ and *xy*‐planes. The chemical system of interest is shown as a green sphere (containing all critical points). The camera is placed −4r along the *z*‐axis, spot‐lights are placed at 45° relative to the origin and a lamp opposite the camera. The key and fill lights are raised r above the *x*‐axis in the *xy*‐plane. [Color figure can be viewed at wileyonlinelibrary.com]

The majority of the program deals with converting topology data as stored in an XML file to its 3D representation, positioning a camera, lighting the scene and customizing the rendering options. When “Import Topology” is clicked, a file select window is presented to the user, who is responsible for navigating to the file of interest. The main part of the program is then executed, as summarized in Figure 6a. The selected file is parsed and individual objects are created for each topological element. Once all records have been parsed, a Topology object is created from the lists of child objects. The program then uses the CP objects to generate a list of the unique materials required for the scene; a corresponding nucleus and surface material is created for each unique element present in the topology (colors are the same as in PyMol), as well as for BCPs (red), RCPs (blue), and CCPs (green). Finally, a single, black AIL material is created. The default materials use the Lambert diffuse shader (intensity 1.0) and the Cook‐Torrance specular shader (white, intensity 0.5). With the topology defined and materials available, the mapping to 3D objects can be carried out. The program iterates over each object of the topology and creates a 3D representation according to the particular type of the object, with materials assigned based on the nature of its associated CP. On completion, the 3D representation of the topology has been created and the mapping is done.

A final function sets up the default world in which the topology is to reside. The purpose of this function is to allow users with no experience of Blender to generate renders without requiring knowledge of the program. First, a camera is created and positioned and then the lights are added (see Fig. 6c). The center of the system $\left(x_{0},y_{0},z_{0}\right)$ is defined as the mean position of all CPs, and its extent r is determined by computing the radius of a sphere placed at the center, which encompasses all the CPs (represented as a green circle in Fig. 6c). The camera is initially placed at the center of the system, facing along the negative *z*‐axis, and is then moved to the position $\left(x_{0},y_{0},-4r\right)$. A basic 3‐point lighting system is used. Key, fill, and rim lights are created and then positioned as shown in the diagram in Figure 6c. The key light is a spotlight added at the camera position pointing toward the system center, and then rotated about the origin by 45 degrees. The process is repeated for the fill light (also a spot light, with lower energy than the key). Both the key and fill lights are raised r above the *xy*‐plane along the *y*‐axis. Finally, a non‐directional lamp to be used as the rim light is added at $\left(x_{0},y_{0},4r\right)$, directly behind the system relative to the camera.

Render settings are then dealt with. The default render resolution ( 1000 × 1000 pixels) is set, anti‐aliasing, a technique for smoothing jagged edges on curved lines (8 samples) is turned on and the pixel filter is set. The output render format is set to .png as default with 16 bits of color depth and no compression. Ray tracing (6 samples) is activated, and finally the background color is changed to sky blue. These choices have been made based on the rendering processes of the figures in the following Results section, and can be easily altered by users through the Blender GUI. With the program is provided a quick‐start guide, which introduces new users to repositioning of the camera and basic functions such as changing the background color.

Along with the import/mapping functionality, Rhorix adds a control panel to Blender (shown in Fig. 6b), which allows the user to read in (and manipulate) a topology. A button to select a topology file is also added to the main menu under File→Import→Quantum Chemical Topology (.top). The simple topology checkbox allows the user to request that the program only read CPs and AILs from the topology file, which can be useful when other line types are not of interest but are present in the file. The Select Nuclei button simply deselects the current selection and selects all nuclei in the system. Render Stereo changes settings related to stereoscopic rendering so that cross‐eyed 3D images are produced (see Results section for details). “Differentiate Interactions” attempts to use built‐in van der Waals radii data to apply different aesthetics to those AILs that would be normally considered “bonds,” and those that would instead be classified as interatomic interactions. This allows easier visual differentiation between the two, which can aid chemical insight. Finally, “Resize AILs” allows the user to move the mouse to scale the AIL radii all at once. Future versions of the program will add more useful tools to this panel to reduce the time required to create images.

The remainder of the Add‐On code is composed of function definitions required for the interface between the script and Blender to work, that is, specification of the Add‐On information and registration of defined functions so that they are available to the user. Other than the main Rhorix Python program, a number of helper Perl libraries and scripts are provided. The simplest of these, “centerTop.pl,” filters the NACPs from a .top file and then moves the entire system such that it becomes centered at the center of mass. This property is useful when a system is clipped from a larger system. The script “terachem2wfn.pl” converts TeraChem output to the ProAIM wavefunction format and is described in the Supporting Information. The remaining scripts all deal with either workflows for execution of AIM analysis programs or conversion of their output to the .top file format. While Rhorix is not discriminatory with respect to the particular scalar function whose topology it depicts, it is necessary to be able to convert the topological data to the described .top format before Rhorix is able to accept it as input. Given the profusion of QCT programs, each with different output formats, it will typically be necessary to write a script to perform this conversion. To mitigate the effort required, a Perl module is provided which contains subroutines for writing .top format files and checking conformity with the document type definition. Conversion scripts for extracting electron density data from MORPHY and AIMAll are also included.

### Computational details

Depicted geometries (other than PSMα3, see Fig. 10) were obtained at the B3LYP^71^, ^72^, ^73^, ^74^/6–31+^75^, ^76^, ^77^G(d)^78^, ^79^ level of theory with the DFT‐D3 dispersion correction^80^ using the Becke‐Johnson damping function.^81^ Geometry optimization was performed with GAUSSIAN09,^82^ and single‐point calculations were used to generate formatted checkpoint files and AIM wavefunction files for QCT analysis with a pruned grid of 99 radial shells and 590 angular points per shell, and the energy convergence was set to $1.0\times 10^{-6}$ Hartrees. Geometry convergence was achieved when the maximum and RMS force were below $1.5\times 10^{-5}$ and $1.0\times 10^{-5}$, respectively, and the maximum and RMS displacement were below $6.0\times 10^{-5}$ and $4.0\times 10^{-5}$, respectively. AIMAll^68^ was used to perform determination of the topology of the electron density, and IRIS was used for triangulation of surfaces. The 3D structures of Figures 2b–2d were generated with the Tachyon ray‐tracer in VMD.^29^, ^83^

An optimized geometry and wavefunction for the model system related to PSMα3 (Fig. 10) was first obtained using TeraChem 1.9^84^, ^85^, ^86^ at the B3LYP/6–311G(d)^77^, ^87^, ^88^//HF/6–311G(d) level of theory, with the particular basis chosen due to the presence of Selenium. The DIIS method of convergence^89^ was used for the HF SCF calculation; all two‐electron integrals were computed with double precision and those less than of $1\times 10^{-13}$ au were neglected. Geometry optimization of this large system was completed in Cartesian coordinates with the maximum energy gradient set to $4.5\times 10^{3}$ au, and the SCF energy change to $1.0\times 10^{-4}$ Hartrees. For DFT calculations, a hybrid DIIS/A‐DIIS^90^ scheme was required to converge the SCF calculation, and a grid of density level 5 (∼80,000 points per atom) was used for final computation of the wavefunction. As this system is too large to generate final checkpoint files with GAUSSIAN09, a Perl script to convert the output to ProAIM format was written, which is detailed in the Supporting Information. Checkpoint and wavefunction files were printed to disk for AIM analysis, again performed with AIMAll. Only bond paths were searched for in the analysis. Default settings were used for all remaining options in all programs. Further details of the QM calculations (construction and stepwise relaxation of the system) can be found in the Supporting Information, along with the final geometry.

Despite the use of the non‐free software Gaussian09 and TeraChem to compute wavefunctions in the described applications, this does not reflect a dependence of Rhorix on these programs. Free software may be substituted provided it produces appropriate input files for a QCT program. The same may be said for AIMAll; Rhorix reads .top files as defined above and it, therefore, does not depend on a particular QCT program, only on the ability to convert the output of a program to .top format. As discussed above, a Perl module for writing .top files is provided with the Add‐On, as are conversion scripts for MORPHY/IRIS and AIMAll.

All QCT visualizations were generated with Rhorix in Blender except Figure 3, made with MORPHY.^60^ Printing of 3D atoms used MeshLab^91^, ^92^ (v1.3.3) to triangulate points on surfaces, and NetFabb (v 7.4.0 532 basic, AutoDesk) was used to correct defects. Meshmixer (v 11.0.544, AutoDesk) was used to thicken surfaces where necessary. Full details are provided in S3 of the Supporting Information.

## Results

**Figure 7:**
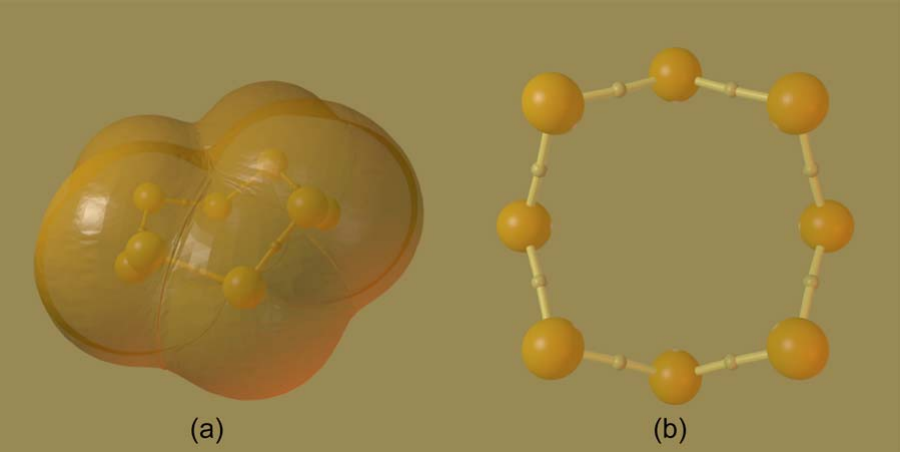
Two images of the S_8_ molecule redrawn from Pauling and Hayward. a) Ball and stick representation with superimposed CPK‐like representation using envelopes of constant electron density ( $\rho \left(r\right)=0.001$ au). b) Ball and stick representation with bond paths and BCPs colored identically, aligned to emphasize the structure as a pair of overlapping squares. [Color figure can be viewed at wileyonlinelibrary.com]

To demonstrate the abilities of Rhorix, a series of interesting chemical systems have been rendered. First, a number of images from “The Architecture of Molecules” a popular science book containing a series of artistic renderings of interesting chemical systems accompanied by Pauling's scientific commentaries are recreated. These images highlight the difference between pre‐computer, empirical representations and QM‐derived, computer‐drawn images of chemical concepts. Figure 7 shows the S_8_ molecule, a cyclic system with a simple molecular graph. In Figure 7a, the molecular graph is depicted along with a set of isosurfaces which enclose roughly 98% of the total electron density. These surfaces stand in for the van der Waals surfaces of the CPK representation but are not exactly spherical in shape. The molecular graph is shown more clearly in Figure 7b where the atomic interaction lines are exposed. The correspondence between the usual view of a bond between atoms and the atomic interaction line is clear; each pair of sulfur atoms is connected through two gradient paths linked to a single mutual BCP. In addition to the automatic lighting, the scene is lit from below with a red light to recreate the effect of the original drawing.

**Figure 8:**
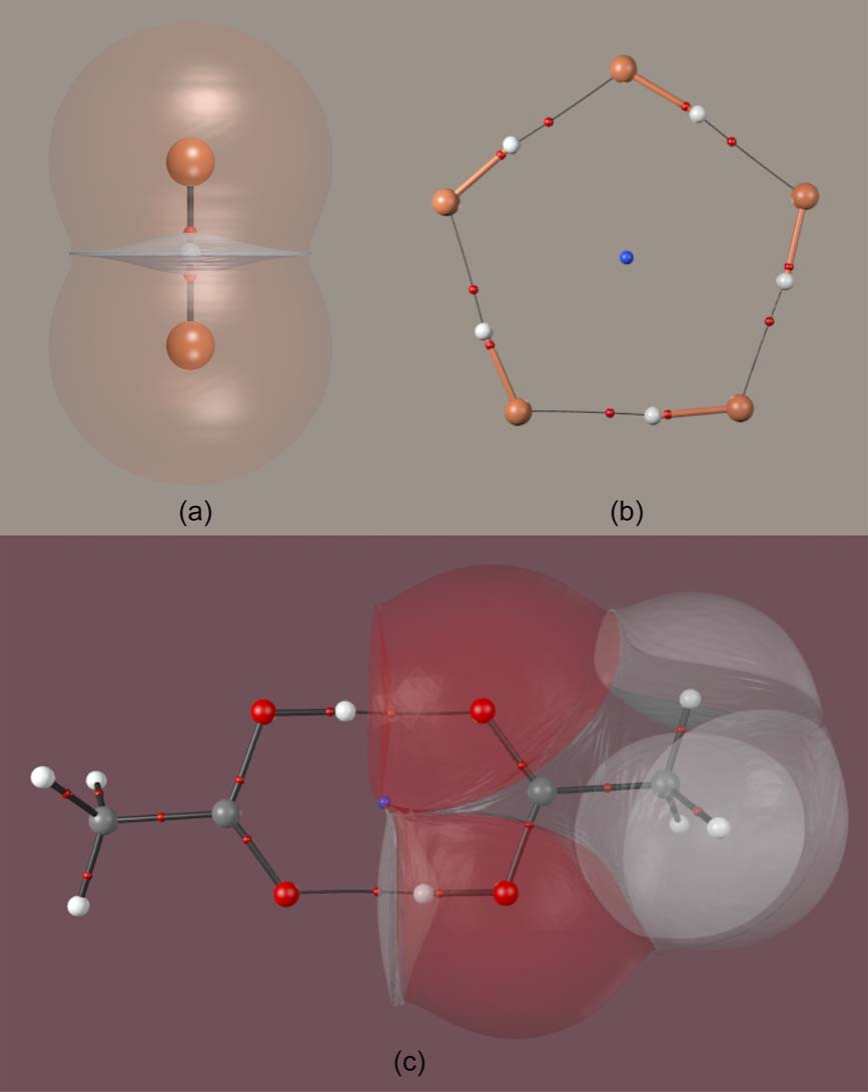
HFH^–^ and systems with hydrogen bonding. a) The HFH^–^ molecule. b) An arrangement of five HF molecules, featuring 10 interactions separable into five identified with HF bonds and five F—H…F identified with hydrogen bonds. An RCP is present at the center of the pentagonal ring. c) The acetic acid dimer, showing a pair of O—H…O hydrogen bonds between monomers. An RCP (blue) is located at the center of the distorted hexagonal ring. [Color figure can be viewed at wileyonlinelibrary.com]

Figure 8 shows renderings of three different systems. The HFH^−^ system is drawn in Figure 8a, with the molecular graph, interatomic surfaces between H and F, and envelopes of the fluorine atoms shown. In this case, all atomic interaction lines are depicted in black, with BCPs in red. The outer envelopes are highly transparent while the interatomic surfaces are less so, with the hydrogen nucleus visible inside. The extremely non‐spherical shape of the hydrogen atom highlights the power of QCT to provide answers from first principles to difficult problems. Figure 8b shows a minimum energy geometry of five HF molecules, each of which donates and receives an interaction that can be interpreted as a hydrogen bond, depicted with a thin black line. Bonds inside HF molecules are shown with thicker cylinders colored according to the element of the NACP they connect to. Topologically there is no difference between the two types of interaction depicted, the differentiation is made purely on the basis of standard chemical intuition. These AILs also constitute a ring, enclosing a surface containing an RCP (in blue, enlarged for emphasis). Figure 8c shows a pair of acetic acid molecules interacting through a pair of strong hydrogen bonds. The outer envelopes of the right‐hand model are depicted to give a sense of molecular size. Again, the representation corresponding to a CPK image shows much more complexity, the O—H…O hydrogen atom having a deeply non‐spherical shape. An RCP is found in the ring formed by the two carboxyl groups.

**Figure 9:**
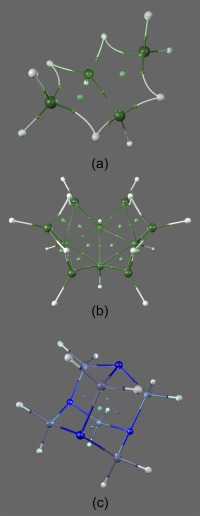
Systems with interesting molecular graphs. a) Tetraborane (B_4_H_10_). b) Decaborane (B_10_H_14_). c) Hexamethylenetetramine (CH_2_)_6_N_4_. In addition to the standard lighting of Figure 6c, these images are lit by light sources at the RCPs (a,b) and CCP (c). [Color figure can be viewed at wileyonlinelibrary.com]

Figure 9 shows three systems with complex molecular graphs, two inorganic borohydrides and an organic amine. Significantly curved atomic interaction lines are seen in the boron‐containing molecules, as well as a large number of RCPs. In Figure 9c, a cage is depicted, with four rings (with corresponding RCPs) enclosing the space containing the CCP. These images show the utility of lighting for highlighting substructures possible with Blender. In Figures 9a and 9b, omnidirectional colored lights are placed at each RCP, while in 9C one is placed at the CCP.

Finally, a large system of recent scientific interest is drawn to highlight the future of topological analysis of scalar fields computed from the wavefunction. Recently, an interesting crystal structure has been reported for an aggregate formed from phenol‐soluble modulin (PSM) proteins, excreted by bacteria and implicated in human disease.^93^ Protein PSMα3, a 22‐residue peptide excreted by *Staphylococcus aureus*, is the most toxic member of the PSM family. Fibrils formed by aggregation of these molecules are of interest as an outlier in known biology. Rather than forming fibrils of β‐strands, they retain their α‐helical conformation on aggregation, forming stacked sheets of helices. Currently, PSMα3 is unique among natural fibrils for this “cross‐α” behavior.

**Figure 10:**
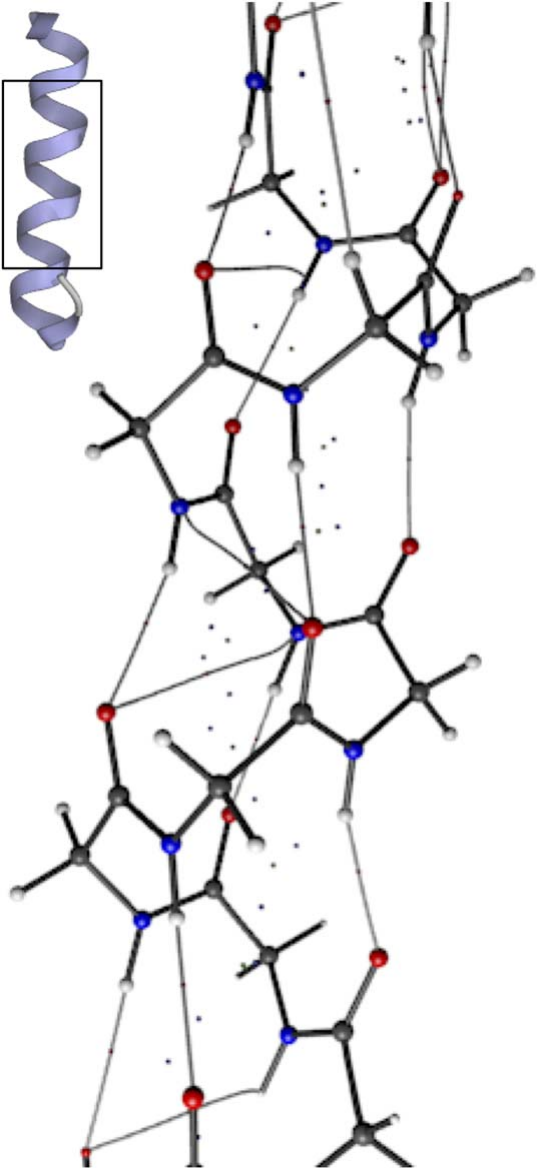
Rendering of the biological assembly of the PSMα3 peptide (PDB 5I55, upper left) with ribbon (residues 1 to 21) and cartoon (residue 22) representations. Interactions inside the helical regions are highlighted via rendering of the topology of the electron density using Rhorix. [Color figure can be viewed at wileyonlinelibrary.com]

Analysis of interactions within helices of the fibrils is a problem well‐positioned for QCT. The presence of 22‐residues in each helix means that this system is of significant size, and is at the edge of systems treatable with quantum chemistry. A single protonated (pH 7.4) fiber (one crystal asymmetric unit) contains 22 residues, or 417 atoms, a significant challenge for modern quantum chemistry. The software TeraChem, specific for GPU computers, is able to treat systems of this size, albeit limited to DFT and relatively small basis sets. However, it has been reported that the topology is little‐affected by basis set and it can be expected that important interactions will be recovered. Figure 10 shows the structure of a single helix, as well as a Rhorix‐generated image of the intra‐helix interactions present in this molecule. For the single helix, the expected N—H…O interactions are present, as well as much longer (and presumably weaker) interactions. QCT, by allowing the computation of gradient paths corresponding to interatomic interactions, can facilitate the study of biological systems of significant size, and allows hypotheses to be made about the relationship between structure and observed phenomena.

### Stereoscopic rendering

**Figure 11:**
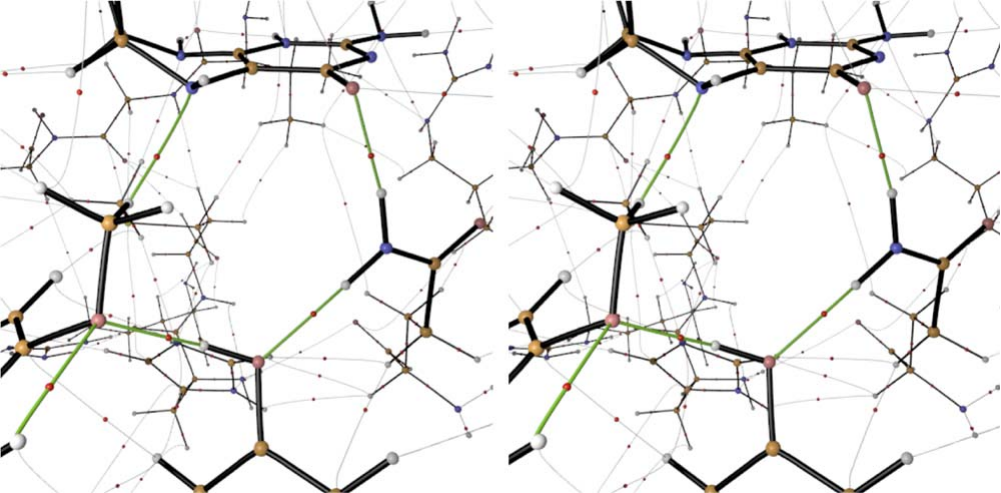
Stereographic image of topology relevant to binding and the reactant state in the catalytic tyrosine‐dependent *O*‐demethylase enzyme LigM. The catalytic tyrosine residue appears at the center bottom of the image. Its proton is transferred to the oxygen atom of the vanillate substrate (lower left) while the substrate methyl group is transferred to N5 of the H_4_folate substrate (top). An asparagine residue (right) interacts with the second substrate and the catalytic tyrosine to complete a cycle of stabilizing interactions. [Color figure can be viewed at wileyonlinelibrary.com]

Even with powerful graphics programs, it can be hard to achieve an appropriate 2D render of a given 3D model. The perception of depth can be difficult to maintain in the rendering process, especially for large, complex systems. It has long been common in biological work to make use of stereoscopic rendering.^94^ However, to the best of our knowledge this method has never been applied to topological figures. Rhorix exposes the stereoscopic rendering feature of Blender, which can assist in difficult cases. Rhorix adds a GUI button (Fig. 6b) to permit creation of a cross‐eyed stereoscopic image of a given topology rather than the standard single flatland image. Two renders with slightly different camera angles are placed side‐by‐side and can be combined by crossing the two pictures by eye movement. An example of such an image, Figure 11, adds stereoscopy to a figure detailing the interactions in the binding of vanillate and tetrahydrofolate substrates in the active site of the enzyme LigM.^95^ The original image was used to determine supporting interactions in place of more usual hydrogen bond maps, which use distances and angles to assign interatomic interactions. A flat 2D render of this image is much less informative as to the 3D geometry of this complicated system.

### Three‐dimensional‐printed atoms

**Figure 12:**
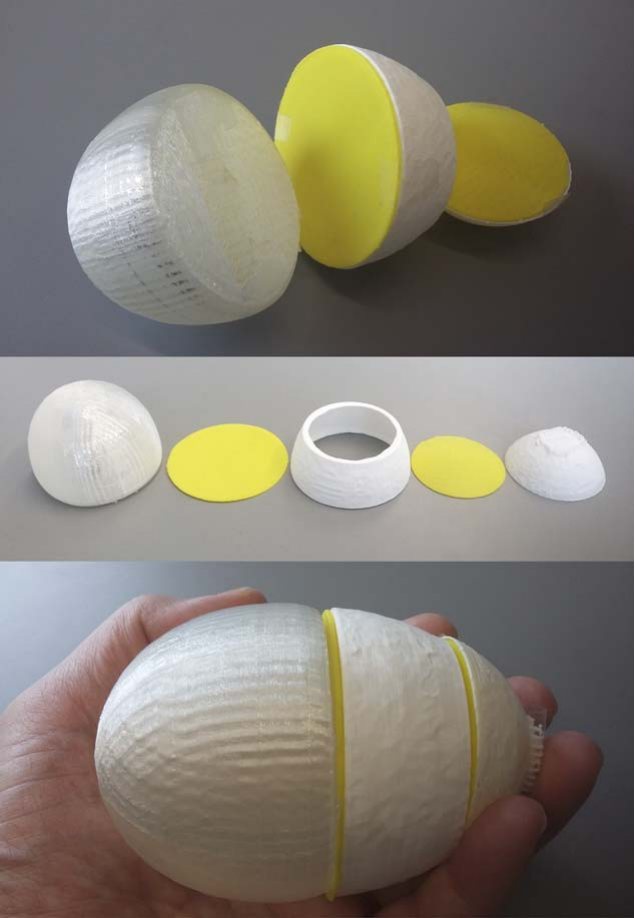
Photographs of 3D printed QCT atoms of the global minimum of the HCN molecule. The top image shows the three atoms, N, C, and H with the carbon atom assembled. The middle panel shows all five pieces separated (including two interatomic surfaces in yellow) and in the final panel, the complete molecule is shown (in hand for scale). [Color figure can be viewed at wileyonlinelibrary.com]

The modern QCT analog of the work of Hoffman, van't Hoff and Corey/Pauling/Koltun discussed in the introduction is 3D‐printed atoms, shown here for the very first time. Several simplifying assumptions of the historical representations are not retained in QCT, specifically the idea of straight‐line bonds and spherical atoms whose shapes are not perturbed by changing conformations. QCT atoms (also called topological atoms) are not necessarily spherical, a property that would be compatible with their space‐filling nature. Thus 3D‐printed QCT atoms are conformation‐dependent. This fact does not prohibit their educational value however, considering how much chemistry education relies on static, single‐conformation representations of molecules. Recently, 3D printed atomic basins for the HCN molecule at its global minimum geometry have been produced, and a set of photographs is provided in Figure 12. Full details of their production can be found in the Supporting Information.

## Conclusion

The described method and implementation provide a simple route to highly detailed rendered images of chemical systems through quantum chemical calculations. Initial work highlights the improvements in aesthetic quality and potential versus existing tools. Preliminary versions created during the development of the described implementation have already been used to generate images for publications.^45^, ^65^, ^66^, ^95^ For the first time, we show the 3D‐printed version of topological atoms, defined according to QCT.

The main barrier to general use of Rhorix is the need for conversion of the output of a given QCT analysis program into the described .top format. A Perl module containing subroutines for writing .top files is currently provided, allowing users to write scripts to use Rhorix with any software or scalar field of interest. In future, it will be advantageous for adoption of the Add‐On to provide users with similar code in a variety of languages, and also to provide scripts specific to various QCT programs and scalar functions.

The current implementation makes use of the Blender render engine, and future versions should take advantage of the more powerful (but more complex) Cycles renderer. In addition, feedback from users will be carefully considered and used to drive improvement of the UI elements added to the Blender control panel. A more distant goal is the development of a separate program for interactive triangulation of surfaces in a graphical interface. Such a program would allow the user to experiment with isocontour values and other triangulation algorithm settings prior to exporting the topology to Blender for rendering.

Finally, the easy generation of movies from 4D data, for example, molecular dynamics trajectories (where the fourth dimension is time) or reaction paths (reaction coordinates), is a very desirable feature. An additional possibility is the introduction of a dynamic element by adding motion of the camera rather than relative motion of the system atoms. This can improve stationary graphics for presentations and online content. Rhorix is available from the Energy Science & Technology Software Center (ESTSC) of the Office of Scientific and Technical Information.

## Supporting information

Supporting Information
