# Supplementary material for: Rhorix: An interface between quantum chemical topology and the 3D graphics program blender
Source: J Comput Chem. 2017 Aug 31;38(29):2538–52. doi: 10.1002/jcc.25054 (PMC5656898; doi:10.1002/jcc.25054)
Supplement: Supplementary file 1 — Supporting Information [file JCC-38-2538-s001.docx]

**Supplementary Material**

**for**

**Rhorix: An Interface Between Quantum Chemical Topology and the 3D Graphics Program Blender**

Matthew J L Mills, Kenneth L Sale, Blake A Simmons and Paul L A Popelier

**1. Preparing TeraChem Output for Atoms in Molecules Analysis**

As discussed in the main text, the methods of quantum chemical topology (QCT) can be used to understand the results of calculations of the wavefunctions of chemical systems in terms of the electron density and other 3D scalar fields. Analysis programs typically require the wavefunction information to be written in the ProAIM .wfn format, although some can read the GAUSSIAN09 formatted checkpoint file, or the extended wavefunction (.wfx) file format. TeraChem, a GPU-leveraging quantum chemistry program, makes quantum chemical calculations on large systems feasible, but does not output any of these wavefunction formats. Therefore file format conversion is required if QCT analyses are to be available. This Appendix describes terachem2wfn.pl, a Perl script included in Rhorix, which carries out the required conversion.

**Background**

Within TeraChem, the wavefunction, $\Psi$, of a chemical system is represented as a set of molecular orbitals, $\phi$, each of which is a linear combination of $N$contracted atomic orbitals, $\varphi$ (AOs, centered at nucleus $A$).

$$\phi\left( \mathbf{r} \right)=\sum_{n=0}^{N-1} c_{n}\varphi_{n}^{A}\left( \mathbf{r} \right)$$

The vector $\mathbf{r}$ has 3 components and specifies electronic spatial coordinates. The set of functions $\varphi_{n}$ is termed the basis set, and is defined prior to calculation of the wavefunction. The coefficients $c_{i}$ are the molecular orbital coefficients, determined within TeraChem by the SCF procedure. Each contracted AO function is a linear combination of $M_{n}$ nuclear-centered Gaussian functions,

$$\varphi_{n}^{A}\left( \mathbf{r} \right)=\sum_{m=0}^{M_{n}-1} d_{m}g_{m}^{A}\left( \mathbf{r} \right)$$

The contraction coefficients $d_{j}$ are fixed for a given basis set, and the Gaussian functions are referred to as primitives. Each primitive is a function of the coordinates of a point $\mathbf{r}=\left\{ x-x_{A}, y-y_{A}, z-z_{A} \right\}$ relative to the atomic center on which the contracted function is centered,

$g_{m}^{A}=N_{m}\left( x-x_{A} \right)^{i}\left( y-y_{A} \right)^{j}\left( z-z_{A} \right)^{k}\exp\left[ -\alpha r^{2} \right]$ (1)

The quantities $i,j,k$ are positive integers. The normalization factor $N$ for each primitive depends on the values of $i,j$ and $k$, and on the orbital exponent, $\alpha$.

$$N=\left( \frac{2\alpha}{\pi} \right)^{3/4}\sqrt{\frac{\left( 8\alpha\right)^{i+j+k}i!j!k!}{\left( 2i \right)!\left( 2j \right)!\left( 2k \right)!}}$$

Although eqn. 1 defines Gaussian primitives in general, they are often discussed in terms of their specific types, determined by the values of $i,j$ and $k$. For $i+j+k=0$ the function is an $s$-type orbital,

$$g_{s}=\left( \frac{2\alpha}{\pi} \right)^{3/4}\exp\left[ -\alpha r^{2} \right]$$

This orbital is spherically symmetric. When $i+j+k=1$ the function is a $p$-type orbital. There are 3 such orbitals: $\left( 1,0,0 \right)$, $\left( 0,1,0 \right)$ and $\left( 0,0,1 \right)$. These orbitals have axial symmetry about a single axis ($x$, $y$ or , respectively).

$$g_{p_{x}}={4\alpha\left( \frac{2\alpha}{\pi} \right)}^{3/4}x\exp\left[ -\alpha r^{2} \right]$$

The final function type found in the basis sets available in TeraChem is the $d$-type orbital, which arises when $i+j+k=2$. There are 6 corresponding combinations of $i, j$ and $k$, each with a different Cartesian prefactor and one of 2 normalization constants.

| $\boldsymbol{i}$ | $\boldsymbol{j}$ | $\boldsymbol{k}$ | Cartesian |
| --- | --- | --- | --- |
| 2 | 0 | 0 | $x^{2}$ |
| 0 | 2 | 0 | $y^{2}$ |
| 0 | 0 | 2 | $z^{2}$ |
| 1 | 1 | 0 | $xy$ |
| 1 | 0 | 1 | $xz$ |
| 0 | 1 | 1 | $yz$ |

**ProAim Wavefunction Format**

The first line of the .wfn file contains the title. Following this line, the number of occupied molecular orbitals, primitive Gaussian functions and nuclei are given, then the elements, Cartesian coordinates and charges of the nuclei. The remainder of the file contains the data specifying the basis set and the molecular orbitals built from it. First the center assignment of each primitive is given, i.e. the 1-indexed integer index of the nucleus at which it is centered. Subsequently the types of each primitive are given in the form of integer codes. The correspondence between these values and the orbital types is given in the following table.

| $\boldsymbol{s}$ | $\boldsymbol{p}_{\boldsymbol{x}}$ | $\boldsymbol{p}_{\boldsymbol{y}}$ | $\boldsymbol{p}_{\boldsymbol{z}}$ | $\boldsymbol{d}_{\boldsymbol{xx}}$ | $\boldsymbol{d}_{\boldsymbol{yy}}$ | $\boldsymbol{d}_{\boldsymbol{zz}}$ | $\boldsymbol{d}_{\boldsymbol{xy}}$ | $\boldsymbol{d}_{\boldsymbol{xz}}$ | $\boldsymbol{d}_{\boldsymbol{yz}}$ |
| --- | --- | --- | --- | --- | --- | --- | --- | --- | --- |
| 1 | 2 | 3 | 4 | 5 | 6 | 7 | 8 | 9 | 10 |

Next the values of the exponent, $\alpha$, of each primitive are printed.

Following the output of the basis set data, the molecular orbitals are written in order of orbital energy. First the orbital’s occupation number and energy are given, then the MO coefficients. These values are written per primitive, and so they incorporate the contraction and normalization coefficients of each primitive along with the coefficient of the contraction that contains that primitive,

$$C_{n,m}=c_{n}d_{m}N_{m}$$

A tag to delineate the end of the MO data is written, after which the total energy and value of the virial are given.

**TeraChem Output**

The first step to conversion is locating all of the necessary information for writing the wavefunction in the TeraChem output files. Version 1.9 of TeraChem added the keyword/value ‘print_post_tc yes’ to enabling printing of the wavefunction information to the file post.tcfchk with high precision. This file contains the nuclear Cartesian coordinates (in units of Bohr), atomic basis set (centers, types, exponents and coefficients), SCF energy and MO coefficients (for the occupied MOs only). Not present in this file are the MO energies and occupation numbers, nor the value of the virial. The MO energies, along with occupation numbers, are written to the file jobname.molden in the calculation’s scratch directory. The virial is not available and so is set to 2.

The basis set is written in order of nuclear centers, i.e. all functions centered on atom 1 are written in the order $s,p,d$ with a line separating each contraction, then functions centered on atom 2 etc. All orbitals are written as a single line, and so it is necessary to print 3 p-type primitives for each p-orbital, and 6 d-type primitives for each d-orbital listed in the basis set. TeraChem lists the MO coefficients per contraction, and hence the order in which p and d-functions are written is important. The order of p-functions is the same as used by the .wfn format, but d-orbitals are in the order xy, xz, yz, xx, yy, zz. When determining the types of the primitives, it is therefore necessary to take care of the ordering, the types being 8, 9, 10, 5, 6, 7.

**2. Creation of Cluster Models**

In order to create the images of interactions in the PSMα3 peptide (see main text Fig. 10), it was necessary to obtain a wavefunction for the relevant piece of the biological assembly. In general, such models need to be made as small as possible so that use of the chosen level of theory is feasible. To simplify the problem, interactions can be separated into intra- and inter-helical, and studied separately. The *intra*-helical interactions (the classic C=O…H-N type) can be recovered without representation of the side chains; the backbone coordinates of a single helix were therefore extracted from the structure in PDB 5I55 using VMD, and the resulting system was protonated at pH 7.4 with Avogadro (for a total charge of 0). No PDB water molecules were retained, and a total of 157 atoms appear in the helix model.

An initial wavefunction was obtained via single point calculation at the HF/6-311G* level of theory using the created geometry. This was followed by optimization of the auto-generated hydrogen positions with all heteroatoms fixed. For the single helix, the geometry was subsequently optimized without constraints, and can be found in Section 4 of this material. The level of theory for evaluation of wavefunctions for AIM analysis was B3LYP/6-311G*; a single point calculation was performed for the system at this level at the optimized geometry. Computational methodology details and citations can be found in the main text.

**3. Details of the 3D Printing Procedure**

The “.mif” file of the HCN molecule contains 5 surfaces (2 interatomic and 3 constant value envelopes) which are made up of thousands of points in the form of xyz-coordinates. For each surface, the software MeshLab (v1.3.3) was used to reconstruct a smooth surface from the points by triangulation. The process generally includes the following steps:

1. The points for each surface are extracted from the mif file to a separate file, and imported into MeshLab as “xyz” format by using “Import mesh”.
2. “Poisson disk sampling” is applied to the imported points with these typical settings: 15,000 for “Set no. of samples“ and the option “base mesh subsampling” enabled.
3. “Compute Normals for Point Sets” is applied with “Neighbour num” set to 16.
4. “Surface Reconstruction: Ball Pivoting” is applied.
5. The reconstructed surface is saved as an “.stl” file.

It is quite common that the reconstructed surface will contain defects, such as holes or overlapped triangles. In this case, the software NetFabb (v 7.4.0 532 basic) can be used to inspect the surface and correct any existing defects. The process of repairing the surface can be summarized by the following:

1. The “.stl” file is opened in NetFabb.
2. The auto “Repair” (red + icon on the tools bar) is applied; defects on the surface are enclosed by yellow lines.
3. Holes on the surface are filled (where present) by using the “Add triangles” tool (green triangle with a small plus sign on the left) by clicking on two sides of an empty triangle.
4. Surface defects due to overlapped triangles are removed (where present) by using the “Select triangle” tool (green triangle). These defects are indicated by regions enclosed by yellow lines. The defects are removed by choosing the “Select triangle” tool and clicking when the mouse is positioned in such a triangle, which will highlight an overlapping triangle in green. The highlighted triangle can be removed by using the “Delete” key.
5. “Apply Repair” is selected after all defects have been dealt with.
6. “Export part” as “STL” is selected to save the repaired surface.

In most cases, the above procedure is sufficient to create a surface that can be 3D printed, but it is possible that the surface produced in this way will not have the required thickness for printing. In this case, the software Autodesk’s “Meshmixer” (version 11.0.544) can be used to thicken the surface.

**4. Optimized Cluster Model Geometry**

| Element | $\boldsymbol{x}$ | $\boldsymbol{y}$ | $\boldsymbol{z}$ |
| --- | --- | --- | --- |
| N | 3.5956519110 | 3.3573978891 | 27.0712208840 |
| C | 3.4736119567 | 4.6922247952 | 26.4066600113 |
| C | 2.4546620098 | 4.5326957286 | 25.2759035585 |
| O | 2.1927198151 | 3.4299953629 | 24.9004169099 |
| N | 1.9369986064 | 5.6670849029 | 24.7959287389 |
| C | 1.2103229595 | 5.6709446103 | 23.5462545113 |
| C | 2.1632479113 | 5.5712707608 | 22.3526348401 |
| O | 3.3505539795 | 5.6291796018 | 22.4995740871 |
| N | 1.5748015170 | 5.4406852410 | 21.1468555675 |
| C | 2.3431907878 | 4.9865421046 | 19.9953402151 |
| C | 3.5168141313 | 5.8845317287 | 19.6232801684 |
| O | 4.5822482588 | 5.4030662799 | 19.3684591672 |
| N | 3.2951198036 | 7.2142281408 | 19.5852452379 |
| C | 4.3250564388 | 8.1098814934 | 19.0919924486 |
| C | 5.5786012491 | 8.1272869797 | 19.9583095837 |
| O | 6.6544519556 | 8.2792050401 | 19.4606503983 |
| N | 5.3914908666 | 8.0004007675 | 21.2885537167 |
| C | 6.5140224609 | 7.9378655809 | 22.1949217143 |
| C | 7.3278139629 | 6.6483046758 | 22.1288269257 |
| O | 8.4869005734 | 6.6612028918 | 22.4293244309 |
| N | 6.6778210605 | 5.5293325494 | 21.7593818943 |
| C | 7.4040947678 | 4.3073880862 | 21.4880280076 |
| C | 8.3278219308 | 4.3924527187 | 20.2741643828 |
| O | 9.4111103384 | 3.8837718943 | 20.3051649370 |
| N | 7.8598213416 | 5.0521099380 | 19.2008071759 |
| C | 8.6926256450 | 5.2341646892 | 18.0308863266 |
| C | 9.9175655387 | 6.1095868655 | 18.2928287708 |
| O | 10.9925762761 | 5.7951456520 | 17.8718414021 |
| N | 9.7125406858 | 7.2278891626 | 19.0161189483 |
| C | 10.8164327175 | 8.1063034126 | 19.3373775130 |
| C | 11.8482454345 | 7.4651210378 | 20.2621431060 |
| O | 13.0196243438 | 7.6342929316 | 20.0723471190 |
| N | 11.3730359236 | 6.7310497067 | 21.2823869160 |
| C | 12.2772661388 | 6.0634976463 | 22.1907800911 |
| C | 13.0844230995 | 4.9440418448 | 21.5390615649 |
| O | 14.2269990017 | 4.7614014685 | 21.8542153821 |
| N | 12.4493623982 | 4.1913323037 | 20.6278545789 |
| C | 13.1532501323 | 3.1564474663 | 19.9069700862 |
| C | 14.2371543425 | 3.6919512989 | 18.9740754870 |
| O | 15.2835893952 | 3.1153799107 | 18.8669789334 |
| N | 13.9530078591 | 4.8163036265 | 18.2958980406 |
| C | 14.9548489213 | 5.4457777083 | 17.4670334510 |
| C | 16.1515411114 | 5.9760640514 | 18.2558542341 |
| O | 17.2666162867 | 5.8313118692 | 17.8396266209 |
| N | 15.8767547753 | 6.6070239212 | 19.4095734932 |
| C | 16.9404480343 | 7.1022475759 | 20.2541254690 |
| C | 17.8368445888 | 5.9969206890 | 20.8114679294 |
| O | 19.0287897548 | 6.1294573082 | 20.8155184464 |
| N | 17.2223579808 | 4.8985007453 | 21.2786913999 |
| C | 17.9938291248 | 3.7960931074 | 21.8065322876 |
| C | 18.8471692709 | 3.0841967911 | 20.7608411277 |
| O | 19.9224220825 | 2.6426856363 | 21.0568046285 |
| N | 18.3318383909 | 2.9745542152 | 19.5247124491 |
| C | 19.1169618365 | 2.4241640644 | 18.4453692594 |
| C | 20.3420638805 | 3.2656611954 | 18.0889425922 |
| O | 21.3708355750 | 2.7386148693 | 17.7681874650 |
| N | 20.1829271086 | 4.5979460602 | 18.1366461690 |
| C | 21.2929773397 | 5.4886733773 | 17.8961903666 |
| C | 22.3848332623 | 5.3989128563 | 18.9601858341 |
| O | 23.5426236208 | 5.3596951846 | 18.6409707181 |
| N | 21.9737219455 | 5.3643843399 | 20.2338793464 |
| C | 22.9326886709 | 5.2628543131 | 21.3143459185 |
| C | 23.7099697226 | 3.9527611593 | 21.2833956304 |
| O | 24.8961573238 | 3.9195163675 | 21.4911645236 |
| N | 23.0108954410 | 2.8433504504 | 20.9914192377 |
| C | 23.6611442100 | 1.5574907850 | 20.9175188423 |
| C | 24.7085543810 | 1.4416455414 | 19.8129507945 |
| O | 25.6511591076 | 0.7152510671 | 19.9493189677 |
| N | 24.4892648149 | 2.1706452348 | 18.7021092350 |
| C | 25.4445931631 | 2.2123884672 | 17.6275227656 |
| C | 26.6547396253 | 3.1259718269 | 17.8194325538 |
| O | 27.6033630780 | 2.9919141735 | 17.0983738125 |
| N | 26.5631630658 | 4.0557040966 | 18.7890216499 |
| C | 27.5839017314 | 5.0472121008 | 19.0079122250 |
| C | 28.3353057443 | 4.9828990802 | 20.3333565678 |
| O | 29.2261656039 | 5.7618235265 | 20.5297699910 |
| N | 27.9493079902 | 4.0560940488 | 21.2267692700 |
| C | 28.5945376941 | 3.9344213964 | 22.5098430177 |
| C | 28.1294420173 | 4.8713669632 | 23.6239545985 |
| O | 28.8774788662 | 5.0981569422 | 24.5346394624 |
| N | 26.8751229943 | 5.3416917710 | 23.5523202108 |
| C | 26.3410187711 | 6.2127052469 | 24.5794729593 |
| C | 24.8361518058 | 6.0222883720 | 24.6930889833 |
| O | 24.1243476326 | 6.3898820212 | 23.7841738676 |
| N | 24.4005174530 | 5.4641213705 | 25.8098171169 |
| C | 23.0097123020 | 5.1746296737 | 26.0960072250 |
| C | 22.8743076873 | 4.2934119930 | 27.3710454562 |
| O | 21.7165487454 | 4.0289286662 | 27.6661647604 |
| O | 23.9320919804 | 3.9770732478 | 27.9150369060 |
| H | 4.4946062988 | 3.2065164915 | 27.4961566122 |
| H | 3.4321825751 | 2.6406288404 | 26.3754634333 |
| H | 2.8945430277 | 3.2308420638 | 27.7822387342 |
| H | 3.1941782283 | 5.4292037052 | 27.1440925247 |
| H | 4.4342812908 | 4.9507124578 | 25.9821669118 |
| H | 2.3196911620 | 6.5292753943 | 25.1053025733 |
| H | 0.5167016817 | 4.8417626277 | 23.5387388448 |
| H | 0.6339188609 | 6.5857194283 | 23.4833182950 |
| H | 0.5955733131 | 5.2767560575 | 21.1241138422 |
| H | 2.7521200071 | 4.0013105670 | 20.1631943324 |
| H | 1.6703496821 | 4.9311854797 | 19.1489118696 |
| H | 2.3675990920 | 7.5514178710 | 19.6713660271 |
| H | 3.9176254355 | 9.1120294831 | 19.0643994680 |
| H | 4.6343044908 | 7.8470081934 | 18.0916933144 |
| H | 4.4892710063 | 7.7609149550 | 21.6179947807 |
| H | 6.1490309510 | 8.0557852431 | 23.2074863723 |
| H | 7.2013971449 | 8.7459998380 | 22.0006636189 |
| H | 5.7354439708 | 5.5895392714 | 21.4638682452 |
| H | 8.0263127842 | 4.0400439576 | 22.3266552082 |
| H | 6.6881361255 | 3.5101992372 | 21.3274894518 |
| H | 6.9289107920 | 5.3925539985 | 19.1960566515 |
| H | 9.0601807638 | 4.2871673363 | 17.6675148867 |
| H | 8.0920869361 | 5.6912969710 | 17.2542886586 |
| H | 8.7848000554 | 7.5212475950 | 19.2103991052 |
| H | 10.4166449579 | 8.9870225290 | 19.8250585948 |
| H | 11.3480420965 | 8.4203151420 | 18.4516456163 |
| H | 10.3974140524 | 6.6839982007 | 21.4525004874 |
| H | 11.6953195070 | 5.6405415590 | 23.0006196786 |
| H | 12.9920889971 | 6.7546071247 | 22.6115319255 |
| H | 11.4828127158 | 4.3269050421 | 20.4537313554 |
| H | 13.6434781514 | 2.4721380976 | 20.5822219360 |
| H | 12.4303641049 | 2.6002049386 | 19.3234045068 |
| H | 13.0452295448 | 5.2134316427 | 18.3429553343 |
| H | 14.4891226981 | 6.2732004355 | 16.9459695177 |
| H | 15.3498248069 | 4.7591071300 | 16.7337943471 |
| H | 14.9344063866 | 6.7692758530 | 19.6742142976 |
| H | 16.4877096656 | 7.6390066525 | 21.0793548839 |
| H | 17.5875088861 | 7.7815709475 | 19.7180932465 |
| H | 16.2325324050 | 4.8727424706 | 21.3455382367 |
| H | 18.6681056370 | 4.1232702265 | 22.5835126341 |
| H | 17.3076442943 | 3.0768994702 | 22.2357963741 |
| H | 17.4014400087 | 3.2669200388 | 19.3449257011 |
| H | 19.4810571734 | 1.4353503870 | 18.6818652625 |
| H | 18.4816224529 | 2.3559555099 | 17.5701173932 |
| H | 19.2956684474 | 4.9827497224 | 18.3556358831 |
| H | 20.9094992703 | 6.5014351511 | 17.8743015584 |
| H | 21.7662622262 | 5.2781643569 | 16.9488421726 |
| H | 21.0125451338 | 5.4911920376 | 20.4481002052 |
| H | 22.4117239696 | 5.3345980823 | 22.2570295485 |
| H | 23.6521538660 | 6.0633121766 | 21.2878210084 |
| H | 22.0205382546 | 2.8857573563 | 20.9340967964 |
| H | 24.1662624739 | 1.3089667232 | 21.8391119058 |
| H | 22.8985987491 | 0.8088850655 | 20.7403742938 |
| H | 23.6547451423 | 2.6995684915 | 18.6228128916 |
| H | 25.8436671809 | 1.2284351986 | 17.4399277144 |
| H | 24.9379052934 | 2.5449406382 | 16.7302857968 |
| H | 25.6825936575 | 4.1974887961 | 19.2230847568 |
| H | 27.1581364226 | 6.0428309355 | 18.9529981304 |
| H | 28.3221240218 | 4.9648432412 | 18.2271589551 |
| H | 27.1857706304 | 3.4628997356 | 21.0091803754 |
| H | 29.6545104250 | 4.1006843643 | 22.4056735876 |
| H | 28.4517685095 | 2.9238047733 | 22.8738090944 |
| H | 26.2932451924 | 5.1014876843 | 22.7822666080 |
| H | 26.5064793535 | 7.2552090418 | 24.3247027846 |
| H | 26.8517997725 | 6.0076583133 | 25.5064221748 |
| H | 25.0290432570 | 5.0853072570 | 26.4824792664 |
| H | 22.5456385776 | 4.6584519453 | 25.2629797742 |
| H | 22.4574373082 | 6.0969398985 | 26.2429328855 |

**Table SI1** – Nuclear elements and Cartesian coordinates (Bohr) for the optimized geometry of the intra-helix interaction model of PSMα3.
